# Bridging the Troponin Blind Window via the miAMI Standard: A Systematic Review and Meta-Analysis of the Circulating MicroRNA-208 Family

**DOI:** 10.3390/medicina62071351

**Published:** 2026-07-13

**Authors:** Augustin Crabbe, Andreea Laura Antohi, Gianina Dodi, Adrian Covic, Samar Abd ElHafeez, Francesco Pesce, Ionut Nistor

**Affiliations:** 1 Faculty of Medicine, Grigore T. Popa University of Medicine and Pharmacy, 700115 Iasi, Romania; antohi.andreea31@yahoo.com (A.L.A.); gianina.dodi@umfiasi.ro (G.D.); adrian.covic@umfiasi.ro (A.C.); ionut.nistor@umfiasi.ro (I.N.); 2 Epidemiology Department, High Institute of Public Health, Alexandria University, Alexandria 21526, Egypt; samarabdelhafeez.epid@gmail.com; 3 Department of Translational Medicine and Surgery, Universita Cattolica del Sacro Cuore, 00168 Rome, Italy; francesco.pesce@fbf-isola.it; 4 Division of Renal Medicine, Ospedale Isola Tiberina-Gemelli Isola, 00168 Rome, Italy

**Keywords:** acute myocardial infarction (AMI), STEMI, NSTEMI, microRNA, meta-analysis, miAMI standard, early diagnosis

## Abstract

*Background and Objectives*: Early diagnosis of acute myocardial infarction (AMI) remains challenging due to the “diagnostic blind window” of conventional protein biomarkers and the limited sensitivity of electrocardiograms in non ST-segment elevation myocardial infarction (NSTEMI). Cardiospecific circulating microRNAs, specifically the microRNA-208 (miR-208) family, have emerged as promising candidates to bridge this gap. This systematic review and meta-analysis evaluated the diagnostic accuracy of circulating miR-208 and outlines a proposed conceptual framework to guide its clinical translation. *Materials and Methods*: PubMed and Embase were systematically searched up to June 24th, 2026, for clinical studies evaluating the diagnostic performance of circulating miR-208a and/or miR-208b against standard reference definitions for AMI. Risk-of-bias assessment using the QUADAS-2 tool was performed independently by two reviewers. Pooled sensitivity and specificity were estimated using bivariate random effects modeling, and sources of heterogeneity were explored via subgroup analyses. *Results*: Forty-one studies enrolling 6306 participants were included in the qualitative synthesis, of which 14 were eligible for meta-analysis. The pooled sensitivity and specificity of circulating miR-208 for AMI detection were 0.89 (95% CI: 0.81–0.94) and 0.90 (95% CI: 0.83–0.94), respectively. Marked between-study heterogeneity was observed. Subgroup analyses revealed significantly higher diagnostic accuracy in isolated STEMI (sensitivity: 0.95) or NSTEMI (sensitivity: 0.93) cohorts compared to mixed chest pain populations (sensitivity: 0.65; *p* < 0.0001). Specificity dropped from 0.90 with healthy controls to 0.80 when using non-AMI controls (*p* = 0.002), indicating spectrum bias. Funnel plots suggested prominent small-study effects. *Conclusions*: Circulating miR-208 exhibits a powerful biological signal for the early detection of cardiomyocyte injury, but its standalone clinical utility is constrained by methodological heterogeneity and publication bias. Rather than an immediate clinical tool, future prospective translation requires evaluating this biomarker within the standardized miAMI framework—conceptually prioritizing future investigation of the hyper-acute (<2 h) window, absolute quantification to resolve normalization variability, and integration into multi-marker point-of-care panels.

## 1. Introduction

Cardiovascular disease remains the leading global cause of mortality, with a disproportionate burden in low- and middle-income countries where healthcare infrastructure and screening programs are underdeveloped [1]. Limited access to care further impairs outcomes: approximately 646 million people worldwide cannot reach a healthcare facility within one hour, particularly in rural and resource-limited settings [2]. Early diagnosis of myocardial infarction is essential, as incremental delays in reperfusion significantly worsen outcomes; specifically, for patients with cardiogenic shock, each 10 min increase in first medical contact-to-device time between 60 and 90 min is associated with a 4% to 7% increase in absolute mortality [3].

Current practice relies on the 12-lead ECG and the detection of high-sensitivity troponins as markers for cardiac infarction. The 2023 ESC Guidelines require ECG acquisition within 10 min of admission to rapidly identify ST-Elevation Myocardial Infarction (STEMI) [4], but sensitivity is limited for NSTEMI and smaller or posterior infarctions [4,5]. Therefore, serial high-sensitivity cardiac troponin (hs-cTn) measurements at 0 h (h) and 1–2 h are recommended for rule-in/rule-out decisions [4]. Creatine kinase-MB (CK-MB) is less useful at this early stage, as it rises only after 4–9 h compared to troponins, which increase within 1–4 h depending on assay sensitivity [5,6]. This timeline creates a blind window in which ruling AMI in or out remains challenging [6].

Furthermore, while both cardiac troponin I (cTnI) and troponin T (cTnT) are predominantly myocardial in origin, cTnT may be re-expressed in certain skeletal muscle diseases, reducing its cardiac specificity but cTnI does not appear to share this limitation [7]. Elevated troponin levels frequently occur in non-ischemic conditions such as anemia, hypotension, renal dysfunction, heart failure, and tachyarrhythmia [5,8]. Aydın et al. additionally highlights chronic renal failure, acute and chronic heart failure, acute pulmonary edema and embolism, COPD, and pulmonary hypertension as further causes of troponin elevation in the absence of AMI [5]. It has also been noted that troponin I may be a more reliable biomarker than troponin T in patients with chronic renal dysfunction [5]. Together, these factors suggest that troponin elevation often reflects myocardial injury rather than infarction, adding uncertainty during early evaluation, especially in patients with atypical presentations or multiple comorbidities [4,5,6,9].

MicroRNAs are short, non-coding RNAs that are remarkably stable in blood, protected from degradation by inclusion in exosomes or protein complexes [10,11]. Notably, certain miRNAs (miR) are enriched in the heart and virtually absent from other tissues, making them attractive markers for cardiac-specific injury detection. The microRNA-208 family (comprising of miR-208a and miR-208b) is encoded in cardiac myosin heavy chain genes and is selectively expressed in cardiomyocytes [12]. Under normal conditions, these miRNAs are barely detectable in circulation, but myocardial injury can lead to their release into the bloodstream. Due to their cardiac specificity any elevation in circulating miR-208 likely reflects cardiomyocyte injury, a concept paralleling troponin release but at the RNA level. Importantly, early studies have suggested that miR-208 (a/b) may rise rapidly after infarction potentially within the first 1–2 h thanks to a leakage mechanism whereas traditional troponin (non-high sensitivity) elevation often requires a longer period [13,14]. Furthermore, miR-208 and other cardiac miRNAs appear in both STEMI and NSTEMI, demonstrating the ability to distinguish AMI in both unstable and stable coronary disease, raising the prospect of a universally applicable marker for acute coronary syndromes [15,16,17]. Although individual isoforms within this family (miR-208a and miR-208b) may exhibit distinct sensitivity profiles due to dynamic cellular shedding, evaluating them collectively under a unified microRNA-208 model provides a more robust overview of this specific cardiospecific genetic locus during acute coronary events.

However, the existing literature remains highly heterogeneous [17,18], as studies differ substantially in design, timing of sample collection, and analytical platforms. Methodological inconsistencies, ranging from “normalization chaos” to the use of healthy volunteers as controls [19,20] or clinically irrelevant comparator groups [21,22,23,24,25], impede direct comparison and prohibit firm conclusions regarding the diagnostic accuracy of circulating miR-208. Given that reperfusion delays during these initial hours carry such a high clinical cost, particularly in high-risk subgroups, a systematic review is crucial to consolidate the evidence base. This work serves as the foundation for the miAMI (microRNA in Acute Myocardial Infarction) framework: a proposed conceptual framework designed to harmonize clinical protocols and multi-marker panels, offering a standardized blueprint to evaluate whether miR-208 can investigatively bridge the “troponin blind window” in early diagnostic decision-making.

## 2. Materials and Methods

This systematic review was registered in the International Prospective Register of Systematic Reviews (PROSPERO: CRD420251180757). Following PRISMA-DTA guidelines, a systematic literature search was conducted in PubMed and Embase up to 24 June 2026 to identify clinical studies evaluating the diagnostic accuracy of circulating microRNA-208 family isoforms (miR-208a/b) against standard clinical reference definitions for acute myocardial infarction. Two independent reviewers performed study selection, risk of bias assessment via the QUADAS-2 tool, and data extraction using a standardized PICO framework. Joint diagnostic accuracy estimates, bivariate random effects modeling (Reitsma approach), and comprehensive subgroup analyses to isolate sources of heterogeneity were performed using RStudio macOS 2025.09.2. (The full extracted study-level diagnostic data is provided in Supplementary Table S1 and search strategy in Note S2).

A detailed, comprehensive description of the eligibility criteria, search strategies, extraction parameters, and explicit statistical models is provided in the Supplementary Materials Section Note S1.

**Figure 1:**
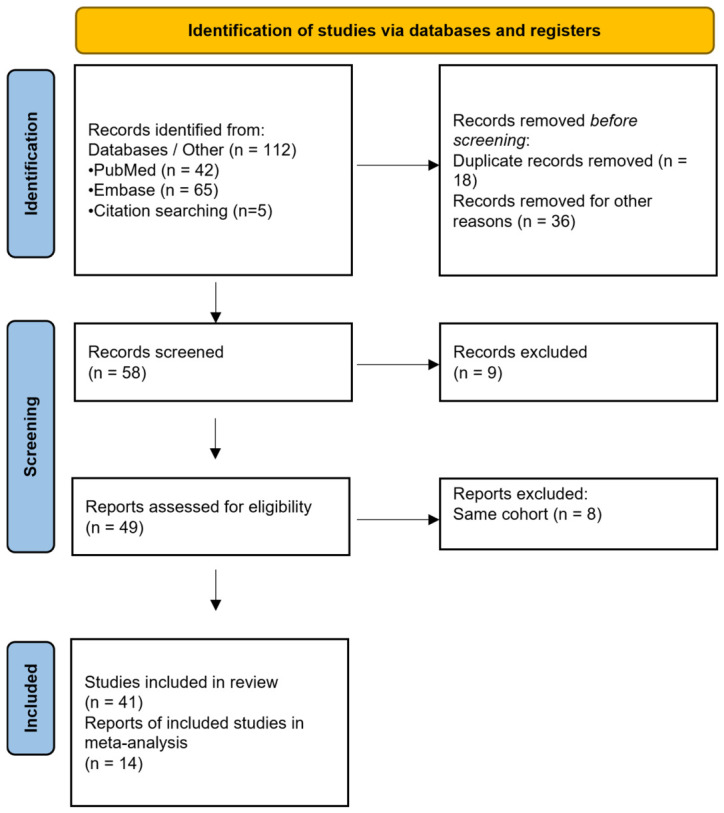
Flow diagram of the study selection process.

The study selection process is summarized in the PRISMA flow diagram (Figure 1), and the full PRISMA checklist is available in Supplementary Table S2.

## 3. Results

### 3.1. Study Characteristics

The last systematic search of PubMed and Embase was conducted through 24th of June 2026. The electronic search identified 112 records (42 from PubMed, 65 from Embase, and five through manual searching). After removal of 18 duplicate records and exclusion of 36 records prior to screening due to clear irrelevance, 58 records remained and were screened based on title and abstract. Nine records were excluded at this stage. Full texts of the remaining 49 reports were assessed eligibility, and a further eight were excluded due to overlapping or identical patient cohorts, often representing preliminary reports by the same authors. Ultimately, 41 studies were included in the qualitative synthesis, of which 14 were eligible for inclusion in the meta-analysis.

### 3.2. Characteristics of Included Studies

**Figure 2:**
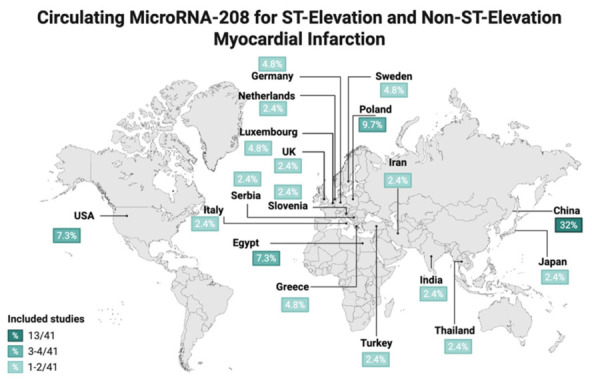
Geographical distribution of studies included in the systematic review.

The 41 studies were published between 2010 and 2025 and together enrolled 6306 participants (range 11–1155). Most studies were conducted in China (13/41), followed by Poland (4/41), the USA (3/41), Egypt (3/41), and individual studies from several European and Asian countries (Germany, Luxembourg, Greece, Italy, Sweden, The Netherlands, Iran, Serbia, Slovenia, India, Thailand, Japan, and the UK) (Figure 2). Most articles used an observational diagnostic or diagnostic case–control design, comparing patients with AMI to heterogeneous control groups ranging from healthy individuals to patients with non-AMI chest pain, acute coronary syndrome, or stable coronary artery disease. A minority focused on prognostic endpoints, kinetic profiling, or post-mortem confirmation of AMI (4/41). Among the 41 included cohorts, 39.0% enrolled patients with unspecified AMI, 31.7% were conducted in STEMI-only populations, 2.4% in NSTEMI-only populations, 14.6% included mixed STEMI/NSTEMI cohorts, and 4.9% enrolled broader ACS populations that included unstable angina.

Regarding clinical presentation, the included AMI populations were highly heterogeneous and often poorly described. Among the 41 studies, 11 enrolled STEMI-only cohorts and two focused exclusively on NSTEMI. Nine studies included both STEMI and NSTEMI patients, although only a minority provided separate analyses for each subtype. A further 17 studies reported AMI cases without differentiating between STEMI and NSTEMI, and two additional studies examined broader acute coronary syndrome (ACS) populations in which myocardial infarction subtypes were not explicitly defined. This inconsistency in reporting substantially limits the ability to perform reliable subtype-specific analyses or to assess whether diagnostic performance differs between STEMI and NSTEMI.

### 3.3. MicroRNA 208 Family and Co-Measured MicroRNAs

All included studies evaluated at least microRNA 208 family (miR-208a and miR-208b). MiR-208a was measured in 14 studies and miR-208b was measured in 20 reports, and it was measured simultaneously in one study. Some studies did not report the type of miR-208 (6/41). Many studies simultaneously quantified other cardiomyocyte-enriched microRNAs (most frequently miR-1, miR-133a, miR-499) to build diagnostic panels or compare the performance of miR-208 against other candidates.

### 3.4. Sample Type, Assay Methods

**Figure 3:**
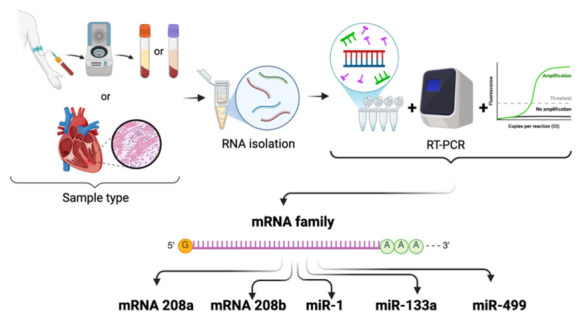
Overview of sample sources and analytical workflow for circulating microRNA-208 detection.

Most studies measured the levels of circulating miR-208 in plasma (25/41) and serum (10/41). A small subset analyzed myocardial tissue or post-mortem blood (4/41) while one study reported a mixed use of plasma and serum, and one [26] used neither plasma nor serum (peripheral blood smear cells) (Figure 3).

Quantification was almost uniformly performed by quantitative reverse transcription PCR (RT-PCR) using commercial kit assays (TaqMan & SYBR, Thermo Fisher Scientific, Waltham, MA, USA) or in-house assays. The internal control varied from endogenous small RNAs (e.g., U6 or miR-16) to other exogenous spike-ins (such as cel-miR-39), and several did not specify their normalization strategy. Cut-off values defining a positive test result were most commonly derived post hoc using ROC curve analysis, with only a minority of studies reporting pre-specified thresholds.

### 3.5. Timing of Sampling

Across the included studies, the timing of blood collection varied greatly from early hours to a day. In most studies, sampling took place upon admission within the first 12 h of chest pain onset. To assess the dynamics of microRNA release, nearly half of the studies used serial sampling schedules, measuring miRNA concentrations at admission and then at several subsequent time points such as 1–3 h, 6 h, 12 h, 24 h and up to 48 h or daily thereafter. Peri-procedural sampling was frequently performed, with blood drawn before and after percutaneous coronary intervention or therapeutic alcohol septal ablation to evaluate the immediate impact of reperfusion [24,27,28,29]. A few case–control studies included post-mortem samples [30,31,32] collected within 12–24 h of death, while others extended follow-up to 5–10 days or even several months. One report uniquely relied on archived samples stored for up to 30 years [33]. Notably, several entries failed to indicate the timing of sampling, highlighting the need for improved consistency in reporting and standardization [19,34,35].

### 3.6. Quality of Included Studies

**Figure 4:**
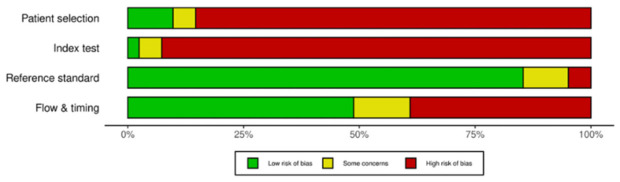
Summary of the QUADAS-2 risk-of-bias assessment across the included studies (n = 41).

Overall, the QUADAS-2 assessment demonstrated substantial methodological limitations across the included studies, with several domains showing a very high risk of bias. The distribution of risk of bias and concerns regarding applicability are summarized in Figure 4, with study-level details provided in Figure S1.

### 3.7. Diagnostic Performance of MicroRNA-208

Based on the extracted data, microRNA-208 (miR-208a and miR-208b) often, but not consistently, showed high discriminatory ability for AMI. When miR-208a was assessed as a single index test, reported AUCs for STEMI diagnosis ranged from 0.918 to 0.9976, with sensitivities of 89–96% and specificities of 80–100% across different cohorts. For miR-208b, several studies indicated near-perfect discrimination: in one STEMI series [36] the AUC was 0.99 with 98% sensitivity and 100% specificity, and another [37] reported an AUC of 1.00 with both sensitivity and specificity at 100%. Very high AUCs (≈0.999–1.000) were also reported when miR-208b was analyzed alongside miR-499 in STEMI patients [18,36], and a multi-miRNA panel including miR-208 achieved an AUC of 0.97 with 93% sensitivity and 98% specificity for differentiating NSTEMI/UA from controls [38]. However, performance was more modest in some datasets: one ACS cohort reported an AUC of 0.57 for miR-208b [23], and other mixed STEMI/NSTEMI cohorts yielded AUCs around 0.67–0.77 with sensitivities of 59.8–82.0% and specificities of 73.6–90.0% [18,26,39,40,41,42]. Composite signatures tended to improve discrimination relative to miR-208 alone. For example, adding another microRNA (miR-499) increased the AUC from 0.76 for miR-208 to 0.911 for the combined model [26]. Several studies specifically demonstrated robust performance in both STEMI and NSTEMI, with miR-208b AUCs of 0.956 and 0.982 in NSTEMI and STEMI [43], respectively, and miR-208a achieving an AUC of 0.965 with 90.9% sensitivity and 100% specificity in another STEMI cohort [14].

## 4. Meta-Analysis

### 4.1. Overall Diagnostic Performance

**Figure 5:**
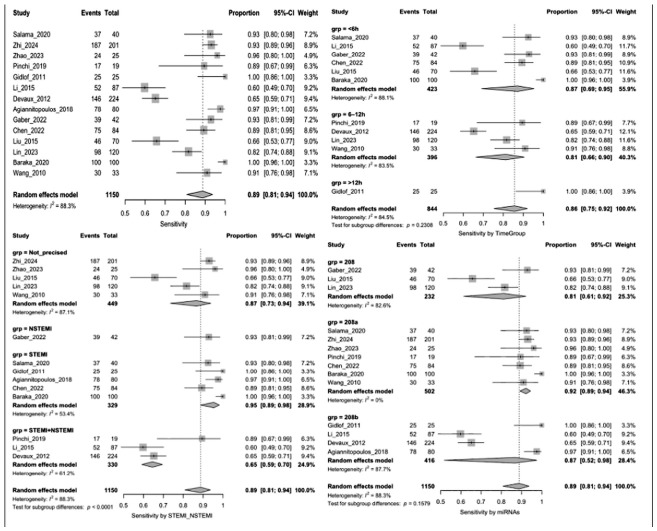
Forest plots showing pooled diagnostic sensitivity of circulating miR-208 family [14,18,19,20,26,27,30,35,36,37,38,39,41,44].

**Figure 6:**
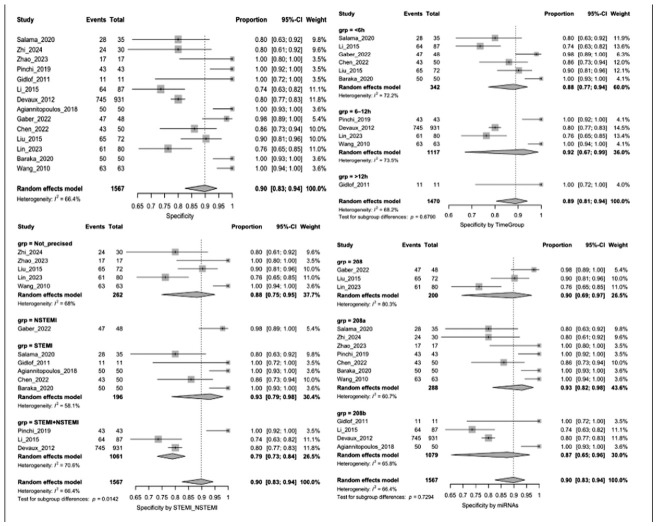
Forest plots of pooled specificity of circulating miR-208 family for the diagnosis of acute myocardial infarction. Individual-study estimates were derived from the included studies [14,18,19,20,26,27,30,35,36,37,38,39,41,44].

Across 14 studies, the pooled sensitivity of circulating miR-208 was 0.89 (95% CI 0.81–0.94) (Figure 5), a wide confidence interval reflecting substantial heterogeneity, and the pooled specificity was 0.90 (0.83–0.94) (Figure 6). The corresponding false-negative rate was 0.11 (0.06–0.19) and the false-positive rate 0.10 (0.06–0.18) (Figure S3). (The complete extracted study-level diagnostic dataset is provided in Table S1 and individual study AUC forest plots are displayed in Figure S5).

### 4.2. Heterogeneity and HSROC

Substantial between-study heterogeneity was observed for sensitivity (I^2^ ≈ 88%) and moderate heterogeneity for specificity (I^2^ ≈ 66%). The HSROC curve showed a summary point close to the pooled sensitivity and specificity, with a wide 95% prediction region, indicating considerable variability across studies (Figure S4).

### 4.3. Subgroup Analyses by miRNA Isoform

Three studies evaluated miR-208 (unspecified isoform), seven evaluated miR-208a, and four evaluated miR-208b, with pooled sensitivities of miR-208: 0.81 (0.61–0.92), miR-208a: 0.92 (0.89–0.94) and miR-208b: 0.87 (0.52–0.98). Specificities ranged from 0.87 to 0.93 across groups. Tests for subgroup differences were not statistically significant (*p* ≈ 0.16 for sensitivity; *p* ≈ 0.73 for specificity). This statistical homogeneity justifies the integration of both transcripts into a singular, comprehensive microRNA-208 diagnostic model, as the minor variations in individual sensitivity profiles do not mathematically distort the overarching diagnostic signal.

### 4.4. Subgroup Analyses by Specimen Type (Figure S2)

Sensitivity estimates were 0.87 (0.72–0.95) for plasma, 0.92 (0.87–0.95) for serum, and 0.83 (0.75–0.88) for other matrices. Specificity was 0.89, 0.92, and 0.93, respectively. The test for subgroup differences in sensitivity approached significance (*p* ≈ 0.053), while specificity differences were not significant (*p* ≈ 0.88).

### 4.5. Subgroup Analyses by Clinical Presentation (Figure 5 and Figure 6)

Sensitivity estimates were STEMI-only cohorts: 0.95 (0.89–0.98); NSTEMI-only: 0.93 (0.81–0.99); AMI not further specified: 0.87 (0.73–0.94); Mixed STEMI + NSTEMI: 0.65 (0.59–0.70). Specificity ranged from 0.79 to 0.98 across groups. Differences were significant for both sensitivity (*p* < 0.0001) and specificity (*p* ≈ 0.014).

### 4.6. Subgroup Analyses by Control Type

Sensitivity was comparable across healthy, non-healthy, and mixed/post-mortem control groups (≈ 0.81–0.91; *p* ≈ 0.89). Specificity differed significantly: healthy controls 0.90 (0.81–0.95) vs. non-healthy controls 0.80 (0.77–0.82) (*p* ≈ 0.002).

### 4.7. Subgroup Analyses by Time to Sampling

Pooled sensitivity was 0.87 (0.69–0.95) when samples were collected <6 h, 0.81 (0.66–0.90) at 6–12 h, and 1.00 (0.86–1.00) after >12 h (one small study) (Figure 5). Specificity varied minimally (≈ 0.88–0.92; *p* ≈ 0.68) (Figure 6). Subgroup differences in sensitivity were not significant (*p* ≈ 0.23).

### 4.8. Publication Bias and Small-Study Effects

Funnel plots for sensitivity and specificity appeared asymmetric, with several small studies reporting high accuracy (Figures S6 and S7). This pattern is consistent with small-study effects.

The predominance of small exploratory studies reporting very high AUC values (frequently >0.95 and occasionally approaching 1.00) suggests possible small-study effects and selective publication of positive findings (Figure S5). Such patterns are characteristic of the early biomarker literature and may contribute to overestimation of true diagnostic performance.

### 4.9. Relationship with Study Quality

Studies judged at lower risk of bias generally exhibited higher diagnostic accuracy estimates [14,40,44,45].

## 5. Discussion

This systematic review was conducted as part of a broader research initiative to evaluate whether circulating microRNAs, specifically the miR-208 family, could serve as rapid, cardiac-specific biomarkers suitable for point-of-care applications, particularly for complex patients with multiple comorbidities.

**Figure 7:**
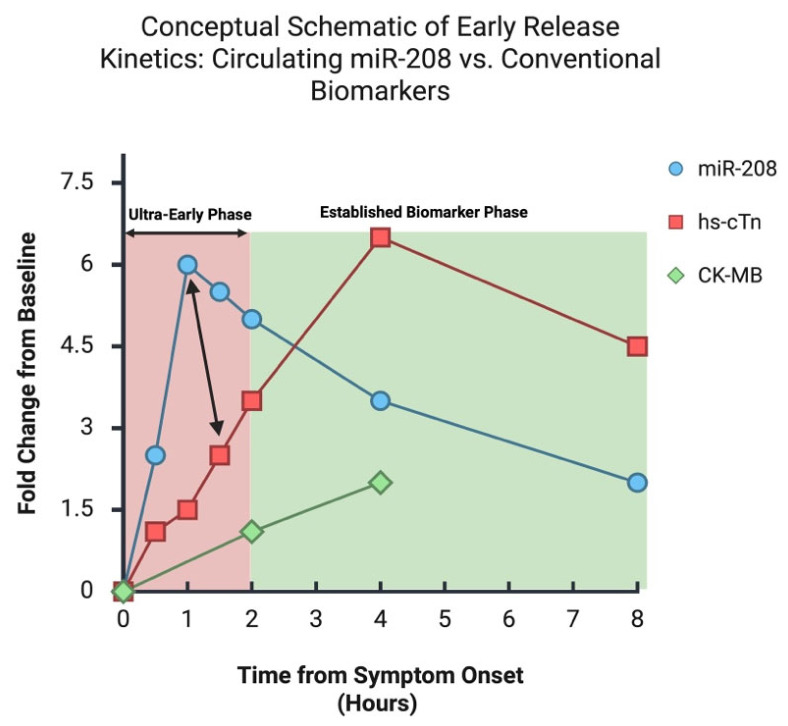
Temporal release profiles of early cardiac biomarkers. Comparative schematic of circulating microRNA-208 (miR-208), high-sensitivity cardiac troponin (hs-cTn) and CK-MB kinetics post-myocardial infarction. The Ultra-Early Phase (0–2 h, shaded red) indicates the initial marker elevation, and the Established Phase (2–8 h, shaded green) denotes the standard clinical evaluation window. The oblique arrow highlights the temporal lead of miR-208 elevation relative to hs-cTn during the ultra-early phase.

MicroRNAs possess several biological features that make them strong candidates for early AMI detection. They are highly stable in circulation, protected from degradation [10,11], and often show strong tissue specificity. MiR-208a and miR-208b are encoded within cardiac myosin heavy chain genes and are minimally detectable in healthy individuals, making their release into the bloodstream a plausible signal of cardiomyocyte injury [12]. As illustrated in our kinetics schematic (Figure 7), miR-208 offers a distinct kinetic advantage, providing a theoretical advantage during the “Ultra-Early Phase” (<2 h) when even high-sensitivity troponins may remain below detection thresholds in the “diagnostic blind spot”.

The main sources of bias were non-rigorous patient selection, healthy controls, and poorly standardized index-test procedures, all of which tend to inflate diagnostic accuracy.

The meta-analysis indicates that circulating miR-208 demonstrates a clear biological signal with pooled sensitivity and specificity close to 0.90, yet these estimates are undermined by substantial heterogeneity across studies. Diagnostic accuracy was markedly higher in narrowly defined STEMI- or NSTEMI-only cohorts, suggesting inflation in selected populations compared with real-world chest-pain presentations, where sensitivity dropped significantly. Variability in specimen type, isoform, sampling window, assay platform, and normalization strategy further contributed to inconsistent performance, particularly in plasma-based studies. Specificity also varied sharply depending on whether controls were healthy volunteers or clinically relevant non-AMI patients, reflecting classic spectrum bias. The asymmetric funnel plots and predominance of small exploratory studies additionally indicated publication bias and the “winner’s curse” phenomenon, where early, underpowered studies report inflated effect sizes that diminish as sample sizes increase. Taken together, these factors show that although miR-208 possesses diagnostic potential, the current evidence base lacks standardization and methodological rigor, preventing reliable translation into clinical practice; at present, miR-208 cannot be recommended as a standalone biomarker for early AMI.

Despite these limitations, the biological specificity of miR-208 remains a compelling advantage over cardiac troponins, particularly in populations with high comorbid burdens. As noted in the introduction, cTnT is frequently chronically elevated in patients with chronic kidney disease (CKD) due to reduced renal clearance and subclinical myocardial injury, complicating the rule-in process. Since miR-208 is exclusively expressed in cardiomyocytes and not skeletal muscle, it theoretically offers an advantage in multi-morbid patients. However, future studies must explicitly stratify performance by renal function (eGFR) to confirm if miR-208 avoids the specificity limitation of troponin utility in nephrology populations.

Several studies demonstrated that miR-208 performed best when evaluated as part of a multi-marker puzzle. Parallel measurements of miR-499 and miR-133a often improved diagnostic performance when incorporated into panels [18,20,36,38]. A combinatorial microRNA signature is more physiologically coherent and diagnostically robust than reliance on a single analyte.

**Figure 8:**
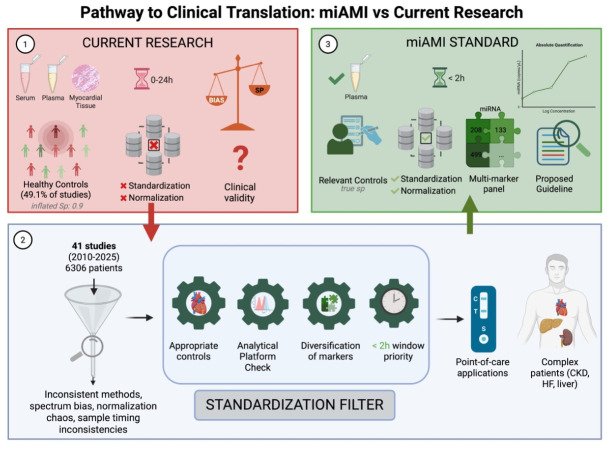
Clinical translation roadmap for microRNA-based diagnostics. Schematic workflow mapping the standardization of microRNA assays from exploratory research to point-of-care application. (1) Current Research: Identification of methodological challenges, including spectrum bias and normalization variability. (2) Standardization Filter: Proposed criteria enforcing clinical controls, analytical validation, and hyper-acute (<2 h) sampling. (3) miAMI Standard: Implementation of absolute quantification and multi-marker panels (miR-208, miR-133, miR-499) optimized for clinical settings, including patients with renal or cardiac comorbidities.

To bridge this gap, we propose the miAMI (microRNA in Acute Myocardial Infarction) framework (Figure 8). This framework acts as a “Standardization Filter” to transition the field from exploratory findings to robust clinical validation by prioritizing:

- Appropriate Controls: Utilizing clinically relevant non-AMI patients to establish true specificity.
- Analytical Platform Check: Moving away from “normalization chaos” toward absolute quantification.
- Diversification of Markers: Developing multi-miRNA diagnostic panels (e.g., miR-133, miR-208, and miR-499) integrated into unified algorithms.
- Ultra-Early Priority: Focusing on the <2 h sampling window to address the critical reperfusion race where every 10 min delay increases mortality risk [3].

Such standardized panels may ultimately offer a more accurate approach to early AMI detection, especially in complex patients with existing heart pathologies or CKD. While not yet feasible for widespread use in resource-limited settings, the transition to the conceptual miAMI Standard may provide a fundamental design blueprint for prospective studies aiming to evaluate whether these assays can be implemented in point-of-care applications within the next few years.

## 6. Conclusions and Future Clinical Feasibility

While the biological specificity of the miR-208 family remains a compelling asset, several pragmatic hurdles must be resolved before clinical integration into emergency department workflows is feasible. Currently, standard quantitative RT-PCR protocols require sample-to-result processing times ranging from 2 to 4 h, largely due to manual RNA extraction phases and specialized thermal cycling. To remain competitive with automated high-sensitivity troponin assays that deliver results in under an hour, future translation relies heavily on the development of microfluidic, extraction-free point-of-care testing (POCT) systems. Furthermore, the current cost per sample of microRNA quantification remains substantially higher than established automated immunoassay chemistries. Finally, unlike cardiac troponins, miR-208 assays remain insufficiently standardized. Inter-laboratory reproducibility is complicated by heterogeneous normalization strategies, including variable endogenous controls such as U6, or mir-16 and exogenous spike-ins such as cel-miR-39. This methodological variability may partly explain the inconsistent diagnostic performance reported across studies.

Transitioning toward digital PCR (dPCR) platforms capable of yielding absolute copy numbers represents a critical component of the proposed miAMI framework to bypass this normalization variability completely. Ultimately, transitioning to this conceptual framework—which mandates absolute quantitative assay platforms, rigorous clinical non-AMI control cohorts, and combinatorial multi-miRNA panels—remains essential to safely evaluate whether the microRNA-208 family can supplement existing clinical algorithms, particularly within complex, high-risk populations where traditional protein biomarkers frequently fail.

## 7. Limitations

This review has several important limitations that reflect the underlying evidence base. Most of the included studies were early exploratory investigations into circulating microRNAs, designed primarily to show differential expression rather than to develop a clinically applicable diagnostic tool. Consequently, methodological rigor and standardization were limited. The studies varied widely in their inclusion criteria (STEMI, NSTEMI, mixed ACS, or not specified), the timing of sample collection, the type of controls used, and the analytical platforms and normalization strategies employed. These inconsistencies limit the direct comparability of the studies, which explains the substantial heterogeneity observed in our pooled sensitivity, specificity, and AUC estimates. This variability does not invalidate the collective signal but underscores that the existing literature was never optimized for reproducibility or translational accuracy, and pooled results must therefore be interpreted with appropriate caution. Furthermore, while the physiological kinetics point to a hyper-acute advantage, our meta-analysis is inherently constrained by the broader sampling intervals (e.g., <6 h) reported in the primary literature. Consequently, the precise diagnostic performance restricted exclusively to the <2 h ultra-early window remains a theoretical framework requiring dedicated, prospective validation.

Additional limitations include restriction to English-language publications, potential omission of unpublished negative studies.

## Data Availability

All data generated or analyzed during this systematic review and meta-analysis are included in this article and its Supplementary Materials. The data were derived from the published studies cited in the article. Further inquiries can be directed to the corresponding author.

## Supplementary Materials

The following supporting information can be downloaded at: https://www.mdpi.com/article/10.3390/medicina62071351/s1, Note S1: Detailed Materials and Methods; Note S2: Full electronic search strategies used for study identification; Figure S1: QUADAS-2 risk of bias assessment for individual studies; Figure S2: Subgroup analyses of diagnostic sensitivity and specificity of circulating miR-208 family by specimen type and analytical platform; Figure S3: False-negative and false-positive rates of circulating miR-208 family for the diagnosis of acute myocardial infarction; Figure S4: Hierarchical summary receiver operating characteristic (HSROC) curve for circulating miR-208 family in the diagnosis of acute myocardial infarction; Figure S5: Forest plot of area under the receiver operating characteristic curve (AUC) for circulating miR-208 family in the diagnosis of acute myocardial infarction; Figure S6: Funnel plot of diagnostic sensitivity (logit scale) for the circulating miR-208 family; Figure S7: Funnel plot of diagnostic specificity (logit scale) for the circulating miR-208 family; Table S1: Full extracted study-level diagnostic data for circulating miR-208 family in acute myocardial infarction. Table S2: PRISMA checklist.
