# Supplementary material for: Bridging the Troponin Blind Window via the miAMI Standard: A Systematic Review and Meta-Analysis of the Circulating MicroRNA-208 Family
Source: Medicina (Kaunas). 2026 Jul 13;62(7):1351. doi: 10.3390/medicina62071351 (PMC13413506; doi:10.3390/medicina62071351)
Supplement: Supplementary file 1 [file medicina-62-01351-s001.zip › medicina-4384296-supplementary.pdf]

Supplementary Material

"Bridging the Troponin Blind Window via the miAMI Standard: A Systematic Review and Meta-Analysis of the Circulating MicroRNA-208 Family"

Augustin Crabbe, Andreea Laura Antohi, Gianina Dodi, Adrian Covic, Samar Abd ElHafeez, Francesco Pesce and Ionut Nistor

Supplementary Note S1: Detailed Materials and Methods

Registration and procedure

This systematic review was registered in the International Prospective Register of Systematic Reviews (PROSPERO) under registration number CRD420251180757.

a) Eligibility Criteria

Eligibility criteria were defined according to the PICO framework. The population comprised adult human participants presenting with suspected acute myocardial infarction (AMI). The index test was circulating microRNA-208 (miR-208a and/or miR-208b) measured in blood-based specimens (plasma, serum, or whole blood). The reference standard was a diagnosis of AMI established according to accepted clinical criteria, typically incorporating cardiac troponin (cTnI or cTnT) measurements and contemporary guideline-based definitions (e.g., Universal Definition of Myocardial Infarction or ESC criteria). The outcomes of interest were diagnostic performance measures, including sensitivity, specificity, accuracy, area under the receiver operating characteristic curve (AUC), and/or reported time to biomarker elevation after symptom onset.

Observational study designs (prospective or retrospective cohort, case–control, and cross-sectional studies) as well as interventional clinical studies reporting diagnostic accuracy data were eligible for inclusion. Only studies published in English from 2010 onwards were considered.

b) Information Sources and Search Strategy

A systematic literature search was conducted in the PubMed and Embase databases up to October 31st, 2025, to identify relevant articles. The search strategy included a combination of Medical Subject Headings (MeSH) and free-text terms. In accordance with PRISMA guidelines, the complete electronic search strategies for all databases are presented in Fig. S1.

c) Study Selection

Two independent reviewers (A.Cr., A.L.A.) screened all titles and abstracts for relevance, followed by full-text assessment for eligibility. No discrepancies were identified.

We used a structured Excel spreadsheet to document the screening process and to organize all extracted variables from the eligible studies. Records retrieved from the searches were first cleaned for obvious duplicates and clearly irrelevant publications, such as non-human studies, papers focused on unrelated microRNAs, or studies not dealing with acute myocardial infarction, before title-abstract screening. Full texts were then assessed against the predefined inclusion and exclusion criteria, and only original human studies reporting circulating microRNAs in the context of AMI with sufficient data for diagnostic evaluation were retained. Studies were excluded at this stage when they relied on overlapping or identical patient cohorts, used exclusively animal or in vitro models, or investigated populations with non-ischemic cardiac conditions

or other non-AMI phenotypes.

The study selection process is summarized in the PRISMA flow diagram (Fig. 1).

d) Data Extraction

Data extraction was performed independently by two reviewers (A.Cr., A.L.A.) using a standardized Excel sheet to ensure completeness and consistency. For each included study, detailed information was collected, including the country of the first author's affiliation, title, author names, year of publication, journal, study design, and database source (PubMed, Embase, or others). The extracted study characteristics comprised the microRNAs investigated (miR-208a, miR-208b, or unspecified 208), the myocardial infarction subtype (STEMI or NSTEMI), and population data such as sample size, mean age, sex distribution, and comorbidities. Data regarding the sample type (plasma, serum, or other), methods of extraction for microRNAs (for example, Trizol™), assay platform or commercial kit used (TaqMan™), timing of sample collection, and recruitment setting were also recorded. Diagnostic performance parameters, including the area under the ROC curve (AUC), sensitivity, specificity, and the reported cut-off values, were extracted along with the reference standard applied in each study, typically cardiac troponin (cTnT/cTnI). Additional information concerning control group characteristics, funding sources, and potential conflicts of interest was noted.

When studies reported multiple sampling time points or multiple thresholds, the earliest clinically relevant sampling window was preferentially selected for primary analysis to reflect the intended early diagnostic context. In cases where only AUC values were reported without explicit 2×2 contingency data, authors were contacted when possible; otherwise, such studies were analyzed separately in an AUC meta-analysis. Reconstruction of contingency tables from reported sensitivity, specificity, and sample sizes was performed assuming internally consistent reporting, recognizing that rounding and post hoc threshold selection may introduce estimation bias

e) Meta-Analysis

All statistical analyses were performed using MacOS RStudio Version 2025.09.2+418 (R version 4.5.2). Meta-analyses followed PRISMA-DTA guidelines. For each study, 2×2 contingency tables were reconstructed using reported sensitivity, specificity, and sample sizes.

Pooled sensitivity and specificity were estimated using a random-effects model of proportions (PLOGIT transformation) with the meta package. Heterogeneity was assessed with I^2 statistics and between-study τ^2. Subgroup analyses were conducted for: miRNA isoform (miR-208, miR-208a, miR-208b), specimen type, STEMI vs NSTEMI, control type (healthy vs non-healthy), time to sample collection (< 6 h, 6–12 h, > 12 h), and type of used platform (SYBR vs TaqMan).

To model diagnostic performance jointly, a bivariate random-effects model (Reitsma approach) was fitted using the mada package, providing pooled estimates of sensitivity, specificity, and the hierarchical summary ROC (HSROC) curve with 95% confidence and prediction regions. The area under the HSROC curve (AUC) and partial AUC were also computed.

Threshold effects were explored by assessing the correlation between logit-transformed sensitivity and logit-transformed false-positive rate. Positive and negative likelihood ratios (LR+ and LR−) and diagnostic odds ratios (DOR) were derived from pooled estimates to facilitate clinical interpretation. Post-test probabilities were estimated using representative pre-test probabilities typical of emergency department chest pain populations. Sensitivity analyses excluding studies at high risk of bias and case–control designs were conducted to evaluate robustness of pooled estimates.

Publication bias was assessed with visual inspection of funnel plots and a Deeks regression test for small-

study effects. Influence analyses (leave-one-out meta-analysis), Baujat plots, and Galbraith (radial) plots were performed to identify studies contributing disproportionately to heterogeneity.

A separate random-effects meta-analysis of AUC values was conducted for studies that reported AUC but did not provide complete Se/Sp data. All statistical tests were two-sided, and $p < 0.05$ was considered significant.

The complete extracted and reconstructed study-level diagnostic dataset is provided in Supplementary Table S7.

f) Risk of Bias Assessment

The risk of bias was assessed using QUADAS-2, following four domains: patient selection, index test, reference standard, and flow & timing. The risk of bias was assessed by two reviewers (A.Cr, A.L.A) and each reviewer completed the assessment independently and recorded justifications for every judgment. Discrepancies between the two reviewers were resolved by discussion. The final domain-level judgments (Low, High, or Unclear risk of bias) were summarized graphically, using the Robvis (Risk-of-Bias Visualization) tool (26) (Figs. 4 and S2).

Particular attention was paid to patient selection methods (consecutive vs case–control sampling), prespecification of index test thresholds, blinding of index test interpretation to reference standard results, and timing consistency between index and reference tests, as these domains are known to substantially influence diagnostic accuracy estimates in biomarker studies.

PubMed Search:

("miRNA-208a" OR "miR-208a" OR "microRNA-208a" OR "miRNA-208b" OR "miR-208b" OR "microRNA-208b") AND ("acute myocardial infarction" OR "AMI" OR "myocardial infarction" OR "heart attack") AND ("diagnosis" OR "diagnostic" OR "biomarker" OR "sensitivity" OR "specificity") AND ("humans" OR "human" OR "human model")

Embase Search:

('microrna-208':ti,ab,kw OR 'microrna 208':ti,ab,kw OR 'mirna-208':ti,ab,kw OR 'mir 208*':ti,ab,kw OR mir208*:ti,ab,kw OR 'mir-208a':ti,ab,kw OR 'mir-208b':ti,ab,kw OR 'hsa-mir-208a':ti,ab,kw) AND 'acute heart infarction'/de AND 'human'

Supplementary Note S2. Full electronic search strategies used for study identification.

Detailed search strings applied in PubMed and Embase for the identification of studies evaluating circulating microRNA-208 family biomarkers in acute myocardial infarction. Searches were conducted up to 31 October 2025 and were limited to human studies published in English.

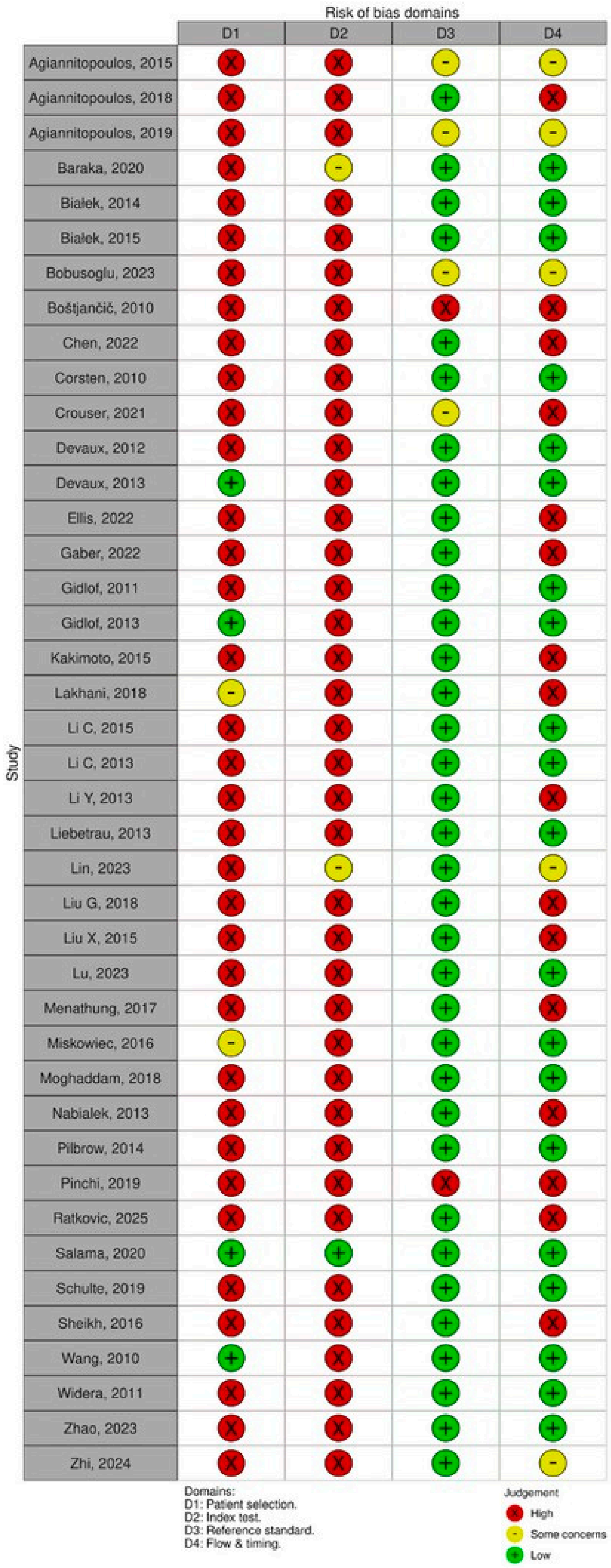

Supplementary Figure S1. QUADAS-2 risk of bias assessment for individual studies.

Traffic-light plot showing study-level risk-of-bias judgments across the four QUADAS-2 domains:

patient selection, index test, reference standard, and flow and timing. Each domain was rated as low risk of bias, high risk of bias, or unclear risk of bias. Assessments were performed independently by two reviewers.

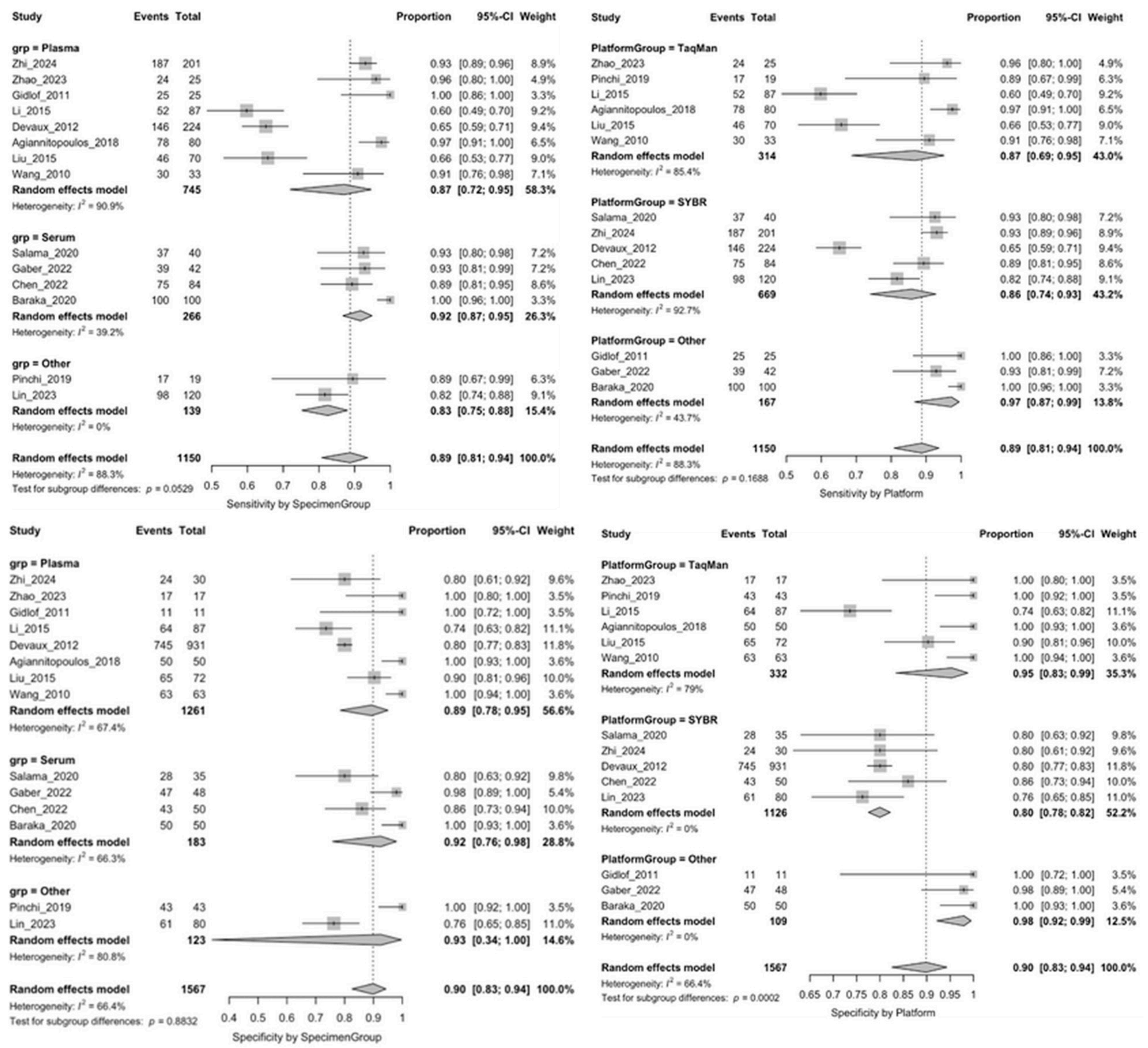

Supplementary Figure S2. Subgroup analyses of diagnostic sensitivity and specificity of circulating miR-208 family by specimen type and analytical platform.

Forest plots show study-specific and pooled estimates of diagnostic sensitivity (top panels) and specificity (bottom panels) with 95% confidence intervals (CI), using random-effects models. Subgroup analyses are stratified by specimen type (plasma, serum, or other biological matrices) and by analytical platform (TaqMan, SYBR, or other methods). Squares represent individual study estimates (size proportional to study weight), horizontal lines indicate 95% CIs, and diamonds represent pooled estimates. Between-study heterogeneity was assessed using the I² statistic, and tests for subgroup differences are reported where applicable.

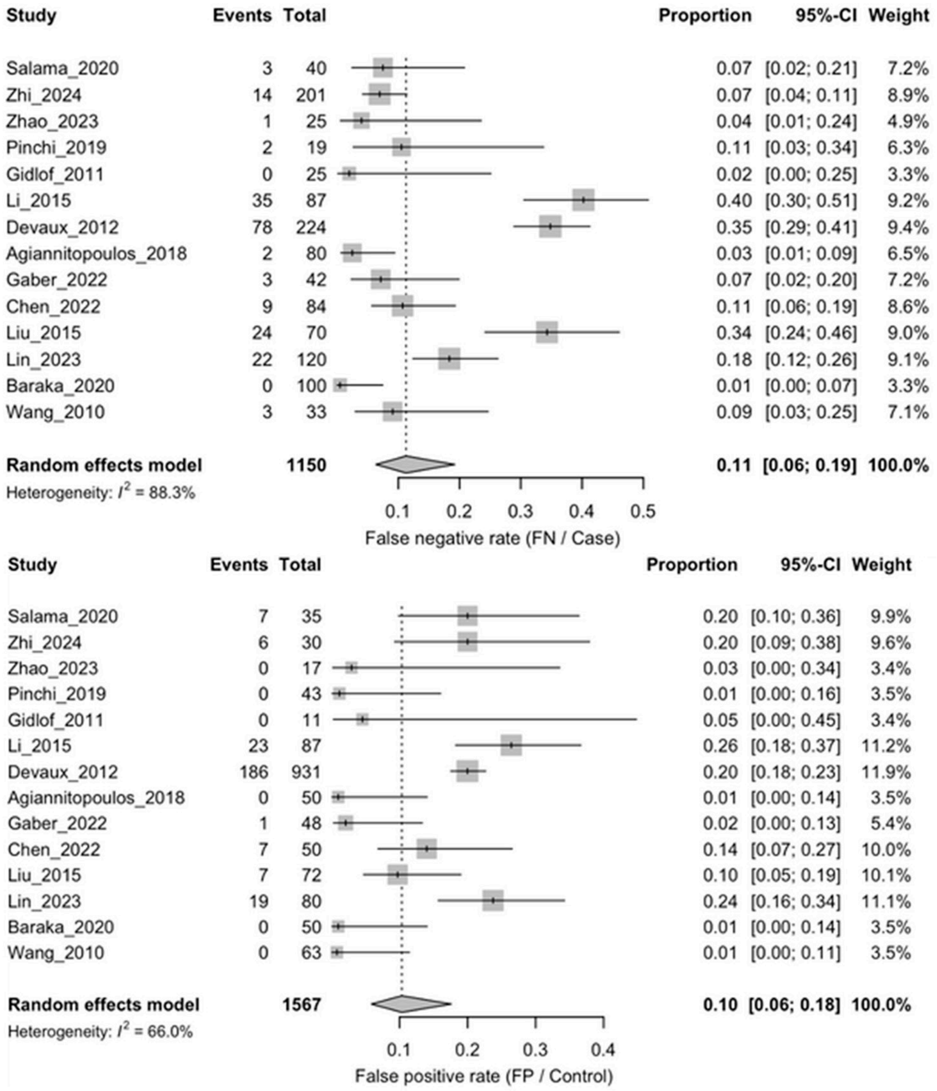

Supplementary Figure S3. False-negative and false-positive rates of circulating miR-208 family for the diagnosis of acute myocardial infarction.

Forest plots show study-specific and pooled estimates of the false-negative rate (top panel; FN / total AMI cases) and false-positive rate (bottom panel; FP / total control subjects) with 95% confidence intervals (CI), calculated using random-effects models. Squares represent individual study estimates (size proportional to study weight), horizontal lines indicate 95% CIs, and diamonds represent pooled estimates. Between-study heterogeneity was assessed using the I^2 statistic.

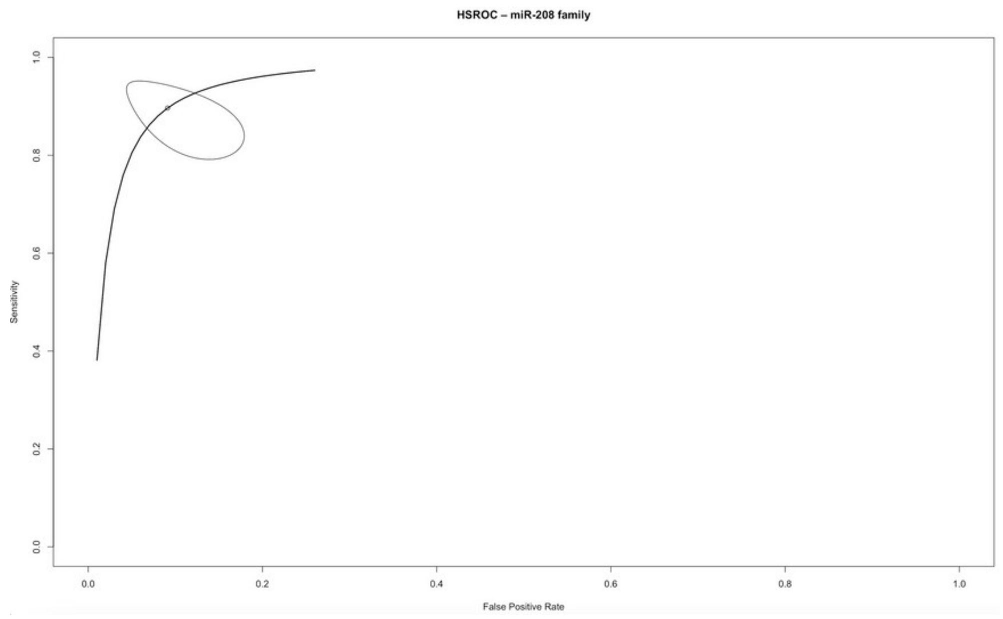

Supplementary Figure S4. Hierarchical summary receiver operating characteristic (HSROC) curve for circulating miR-208 family in the diagnosis of acute myocardial infarction.

The HSROC curve was generated using a bivariate random-effects model, jointly summarizing sensitivity and specificity across included studies. The summary operating point represents the pooled sensitivity and specificity, with the surrounding ellipse indicating the 95% confidence region and the outer ellipse the 95% prediction region, reflecting between-study heterogeneity.

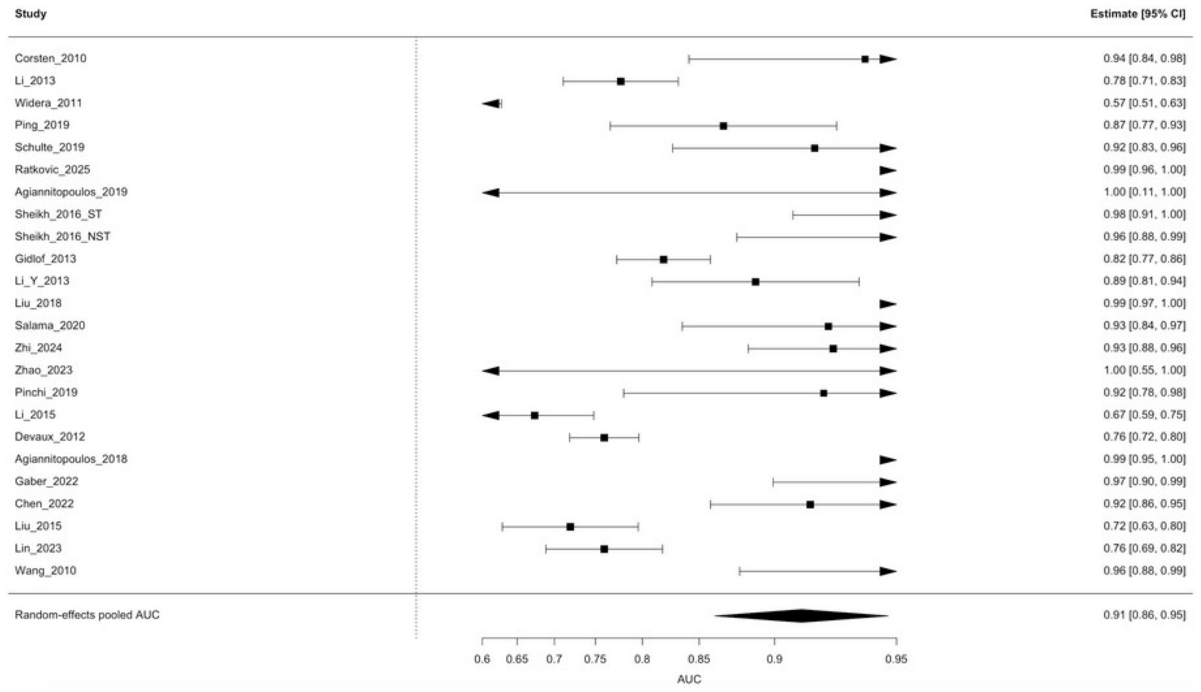

Supplementary Figure S5. Forest plot of area under the receiver operating characteristic curve (AUC) for circulating miR-208 family in the diagnosis of acute myocardial infarction.

Individual study AUC estimates with corresponding 95% confidence intervals are shown for all studies reporting AUC values, including those not eligible for inclusion in the bivariate sensitivity–specificity meta- analysis due to incomplete 2×2 diagnostic data. The pooled AUC was calculated using a random-

effects model, and the diamond represents the summary estimate with its 95% confidence interval. Arrowheads indicate confidence intervals extending beyond the plotted range.

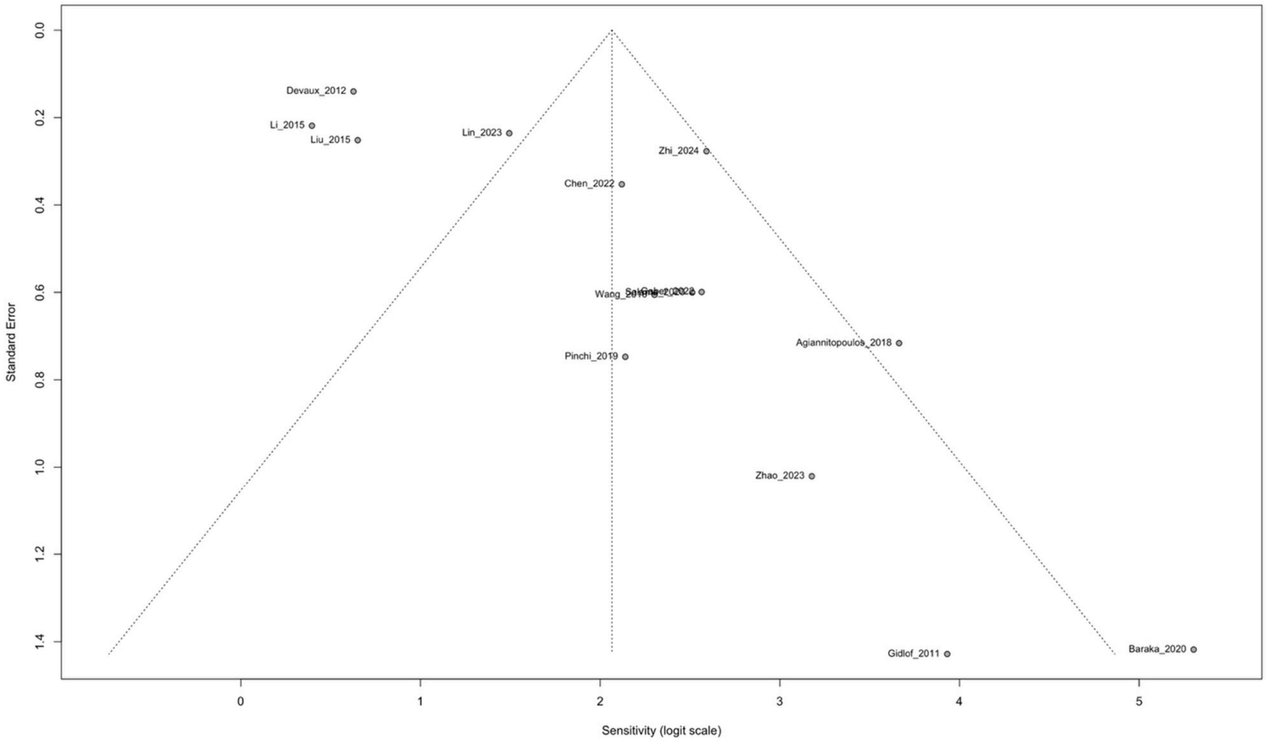

Supplementary Figure S6: Funnel plot of diagnostic sensitivity (logit scale) for the circulating miR-208 family. Deeks' linear regression asymmetry test was utilized to quantitatively assess small-study effects and publication bias across the meta-analysis cohort. The noticeable asymmetry and clustering of smaller exploratory studies outside the pseudo-95% confidence intervals indicate potential publication bias and overestimation of diagnostic performance characteristic of early biomarker literature.

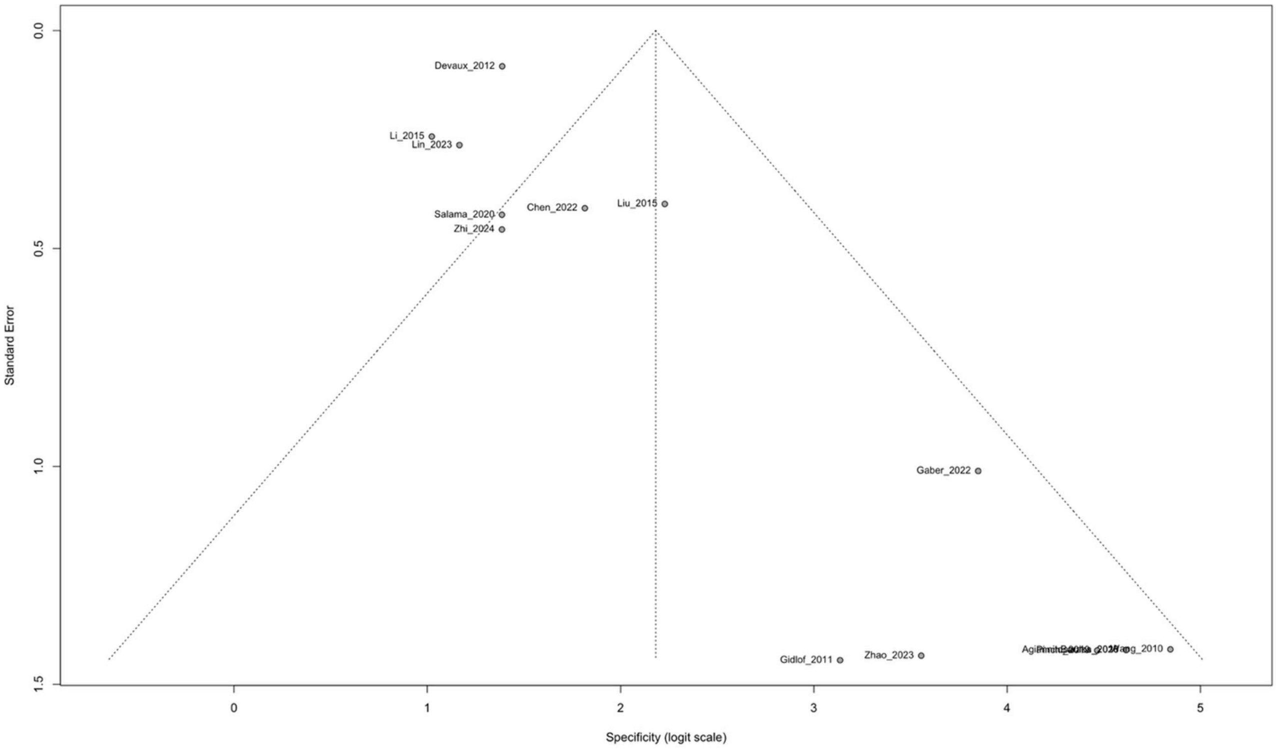

Supplementary Figure S7: Funnel plot of diagnostic specificity (logit scale) for the circulating miR-208 family. Visual inspection of the funnel plot demonstrates marked asymmetry, confirming the presence of selective reporting and small-study effects. Precise clustering of specific datasets on the left boundary indicates how spectrum variations between healthy volunteers and clinically relevant non-AMI controls introduce bias into raw specificity estimates.

Supplementary Table S1. Full extracted study-level diagnostic data for circulating miR-208 family in acute myocardial infarction.

This table summarizes all study-level data extracted from the included publications evaluating circulating miR- 208 (miR-208, miR-208a, or miR-208b) for the diagnosis of acute myocardial infarction. Variables include myocardial infarction subtype (STEMI, NSTEMI, or mixed), sample size, specimen type, control group characteristics, analytical platform, timing of blood sampling, and reported diagnostic performance metrics (AUC, sensitivity, specificity). True-positive (TP), false-negative (FN), false-positive (FP), and true-negative (TN) values were extracted directly when available or reconstructed from reported sensitivity, specificity, and sample sizes for use in meta-analyses.

	Author	miRNAs	STEMI_NSTEMI	Case	Control	Specimen	ControlType	Platform	Time_to_sample	TimeGroup	AUC	Sensitivity	Specificity	TP	FN	FP	TN
1	Gaber_2022	208	NSTEMI	42	48	Serum	Healthy	Other	<6h	<6h	0.9700	0.9300	0.9800	39	3.0	1.0	47
2	Lin_2023	208	Not_precised	120	80	Blood	Healthy	SYBR	<12h	6–12h	0.7600	0.8200	0.7685	98	22.0	19.0	61
3	Liu_2015	208	Not_precised	70	72	Plasma	Healthy	TaqMan	<6h	<6h	0.7200	0.6500	0.9000	46	24.0	7.0	65
4	Baraka_2020	208a	STEMI	100	50	Serum	Healthy	NR	<6h	<6h	1.0000	1.0000	1.0000	100	0.5	0.5	50
5	Chen_2022	208a	STEMI	84	50	Serum	Healthy	SYBR	<6h	<6h	0.9180	0.8928	0.8600	75	9.0	7.0	43
6	Pinchi_2019	208a	STEMI+NSTEMI	19	43	Tissue	Postmortem	TaqMan	<12h	6–12h	0.9240	0.9170	1.0000	17	2.0	0.5	43
7	Salama_2020	208a	STEMI	40	35	Serum	Non-healthy	SYBR	<6h	<6h	0.9260	0.9250	0.8000	37	3.0	7.0	28
8	Wang_2010	208a	Not_precised	33	63	Plasma	Mixed	TaqMan	<12h	6–12h	0.9650	0.9090	1.0000	30	3.0	0.5	63
9	Zhao_2023	208a	Not_precised	25	17	Plasma	Healthy	TaqMan	NR	NA	0.9976	0.9600	1.0000	24	1.0	0.5	17
10	Zhi_2024	208a	Not_precised	201	30	Plasma	Healthy	SYBR	NR	NA	0.9280	0.9320	0.8000	187	14.0	6.0	24
11	Agiannitopoulos_2018	208b	STEMI	80	50	Plasma	Healthy	TaqMan	<24h	NA	0.9900	0.9800	1.0000	78	2.0	0.5	50
12	Devaux_2012	208b	STEMI+NSTEMI	224	931	Plasma	Non-healthy	SYBR	<12h	6–12h	0.7600	0.6500	0.8000	146	78.0	186.0	745
13	Gidlof_2011	208b	STEMI	25	11	Plasma	Healthy	Other	>12h	>12h	1.0000	1.0000	1.0000	25	0.5	0.5	11
14	Li_2015	208b	STEMI+NSTEMI	87	87	Plasma	Healthy	TaqMan	<6h	<6h	0.6740	0.5980	0.7360	52	35.0	23.0	64

Supplementary Table S2. PRISMA checklist.

Section and Topic	Item #	Checklist item	Location where item is reported
**TITLE**			
Title	1	Identify the report as a systematic review.	1
**ABSTRACT**			
Abstract	2	See the PRISMA 2020 for Abstracts checklist.	1
**INTRODUCTION**			
Rationale	3	Describe the rationale for the review in the context of existing knowledge.	2
Objectives	4	Provide an explicit statement of the objective(s) or question(s) the review addresses.	3
**METHODS**			
Eligibility criteria	5	Specify the inclusion and exclusion criteria for the review and how studies were grouped for the syntheses.	3 & supplementary
Information sources	6	Specify all databases, registers, websites, organisations, reference lists and other sources searched or consulted to identify studies. Specify the date when each source was last searched or consulted.	“
Search strategy	7	Present the full search strategies for all databases, registers and websites, including any filters and limits used.	“
Selection process	8	Specify the methods used to decide whether a study met the inclusion criteria of the review, including how many reviewers screened each record and each report retrieved, whether they worked independently, and if applicable, details of automation tools used in the process.	“
Data collection process	9	Specify the methods used to collect data from reports, including how many reviewers collected data from each report, whether they worked independently, any processes for obtaining or confirming data from study investigators, and if applicable, details of automation tools used in the process.	“
Data items	10a	List and define all outcomes for which data were sought. Specify whether all results that were compatible with each outcome domain in each study were sought (e.g. for all measures, time points, analyses), and if not, the methods used to decide which results to collect.	“
	10b	List and define all other variables for which data were sought (e.g. participant and intervention characteristics, funding sources). Describe any assumptions made about any missing or unclear information.	“
Study risk of bias assessment	11	Specify the methods used to assess risk of bias in the included studies, including details of the tool(s) used, how many reviewers assessed each study and whether they worked independently, and if applicable, details of automation tools used in the process.	“
Effect measures	12	Specify for each outcome the effect measure(s) (e.g. risk ratio, mean difference) used in the synthesis or presentation of results.	“
Synthesis methods	13a	Describe the processes used to decide which studies were eligible for each synthesis (e.g. tabulating the study intervention characteristics and comparing against the planned groups for each synthesis (item #5)).	“
	13b	Describe any methods required to prepare the data for presentation or synthesis, such as handling of missing summary statistics, or data conversions.	“

Section and Topic	Item #	Checklist item	Location where item is reported
	13c	Describe any methods used to tabulate or visually display results of individual studies and syntheses.	"
	13d	Describe any methods used to synthesize results and provide a rationale for the choice(s). If meta-analysis was performed, describe the model(s), method(s) to identify the presence and extent of statistical heterogeneity, and software package(s) used.	"
	13e	Describe any methods used to explore possible causes of heterogeneity among study results (e.g. subgroup analysis, meta-regression).	"
	13f	Describe any sensitivity analyses conducted to assess robustness of the synthesized results.	"
Reporting bias assessment	14	Describe any methods used to assess risk of bias due to missing results in a synthesis (arising from reporting biases).	"
Certainty assessment	15	Describe any methods used to assess certainty (or confidence) in the body of evidence for an outcome.	N/A
**RESULTS**			
Study selection	16a	Describe the results of the search and selection process, from the number of records identified in the search to the number of studies included in the review, ideally using a flow diagram.	4 (Figure 1)
	16b	Cite studies that might appear to meet the inclusion criteria, but which were excluded, and explain why they were excluded.	4
Study characteristics	17	Cite each included study and present its characteristics.	4-5
Risk of bias in studies	18	Present assessments of risk of bias for each included study.	6-7
Results of individual studies	19	For all outcomes, present, for each study: (a) summary statistics for each group (where appropriate) and (b) an effect estimate and its precision (e.g. confidence/credible interval), ideally using structured tables or plots.	9-10
Results of syntheses	20a	For each synthesis, briefly summarise the characteristics and risk of bias among contributing studies.	6 (figure 4)
	20b	Present results of all statistical syntheses conducted. If meta-analysis was done, present for each the summary estimate and its precision (e.g. confidence/credible interval) and measures of statistical heterogeneity. If comparing groups, describe the direction of the effect.	7-8
	20c	Present results of all investigations of possible causes of heterogeneity among study results.	8
	20d	Present results of all sensitivity analyses conducted to assess the robustness of the synthesized results.	7-8
Reporting biases	21	Present assessments of risk of bias due to missing results (arising from reporting biases) for each synthesis assessed.	8
Certainty of evidence	22	Present assessments of certainty (or confidence) in the body of evidence for each outcome assessed.	9
**DISCUSSION**			
Discussion	23a	Provide a general interpretation of the results in the context of other evidence.	10-12
	23b	Discuss any limitations of the evidence included in the review.	13
	23c	Discuss any limitations of the review processes used.	13
	23d	Discuss implications of the results for practice, policy, and future research.	12-13
**OTHER INFORMATION**			

Section and Topic	Item #	Checklist item	Location where item is reported
Registration and protocol	24a	Provide registration information for the review, including register name and registration number, or state that the review was not registered.	3
	24b	Indicate where the review protocol can be accessed, or state that a protocol was not prepared.	3
	24c	Describe and explain any amendments to information provided at registration or in the protocol.	N/A
Support	25	Describe sources of financial or non-financial support for the review, and the role of the funders or sponsors in the review.	13
Competing interests	26	Declare any competing interests of review authors.	13
Availability of data, code and other materials	27	Report which of the following are publicly available and where they can be found: template data collection forms; data extracted from included studies; data used for all analyses; analytic code; any other materials used in the review.	13

From: Page MJ, McKenzie JE, Bossuyt PM, Boutron I, Hoffmann TC Mulrow CD, et al. The PRISMA 2020 statement: an updated guideline for reporting systematic reviews. BMJ 2021;372:n71. doi: 10.1136/bmj.n71
